# Hints on the Increase of Hydrocephalus Internus

**Published:** 1818-06

**Authors:** 


### *Hints on the Increase of Hydrocephalus Internus*; by R. N. S. Surgeon, of Barnsley.

THE frequent occurrence of Hydrocephalus Internus, more particularly amongst children of the lower class, I have repeatedly observed; the majority of which cases occur in children under two years of age. This has often induced me to enquire more particularly into the treatment of those children. It appears, from my observations, that, out of four cases of the disease, three have been in the habit of receiving laudanum from their mothers or nurses. This pernicious custom is more generally practised in manufacturing districts, where the female population have employment. By these means, the unfortunate sufferer is kept almost in a state of stupor. We are all aware of the effects of that remedy upon the brain. A common medicine, which is retailed by the druggists, and sold under the name of Child's Cordial, consists of opium, treacle, and water; in the proportion of one grain of the former to an ounce of the latter.

The quantity of this remedy consumed in the manufacturing districts is astonishing: I have heard of one druggist retailing the quantity of eight gallons per week!

When a practitioner is called to a child labouring under symptoms of hydrocephalus, I should recommend his asking the mother, or nurse, whether they have been in the habit of giving the child any composition of this kind; for I have frequently observed the insensibility to stimuli which exists in cases where opium has been given. I am afraid it will be found a more frequent occurrence than is generally imagined. It may be said, that the usual symptoms of hydrocephalus resemble those that take place where opium has been given,—this may be true; but, as before remarked, that peculiar want of susceptibility to the action, even of powerful remedies, is most generally attendant on these cases.

By a careful observation of these circumstances, the practitioner will be able to combat this most formidable disease, by being acquainted with its proximate cause.

*April* 18, 1818.

---

*** Our readers will recollect a similar remark in one of our "Reports," where we hazarded a conjecture that the increase of hydrocephalus might be in part owing to the quantity of porter which mothers and wet-nurses conceive it necessary to take. The composition of porter is not accurately ascertained; but supposing it to be a legitimate mixture of malt and hops, when taken to excess, the child may suffer in that most important organ, the brain.—Edit.

